# *In silico* analysis enabling informed design for genome editing in medicinal cannabis; gene families and variant characterisation

**DOI:** 10.1371/journal.pone.0257413

**Published:** 2021-09-22

**Authors:** L. Matchett-Oates, S. Braich, G. C. Spangenberg, S. Rochfort, N. O. I. Cogan

**Affiliations:** 1 Agriculture Victoria, AgriBio, The Centre for AgriBioscience, Bundoora, Victoria, Australia; 2 School of Applied Systems Biology, La Trobe University, Bundoora, Victoria, Australia; Beni Suef University Faculty of Veterinary Medicine, EGYPT

## Abstract

**Background:**

Cannabis has been used worldwide for centuries for industrial, recreational and medicinal use, however, to date no successful attempts at editing genes involved in cannabinoid biosynthesis have been reported. This study proposes and develops an *in silico* best practices approach for the design and implementation of genome editing technologies in cannabis to target all genes involved in cannabinoid biosynthesis.

**Results:**

A large dataset of reference genomes was accessed and mined to determine copy number variation and associated SNP variants for optimum target edit sites for genotype independent editing. Copy number variance and highly polymorphic gene sequences exist in the genome making genome editing using CRISPR, Zinc Fingers and TALENs technically difficult. Evaluation of allele or additional gene copies was determined through nucleotide and amino acid alignments with comparative sequence analysis performed. From determined gene copy number and presence of SNPs, multiple online CRISPR design tools were used to design sgRNA targeting every gene, accompanying allele and homologs throughout all involved pathways to create knockouts for further investigation. Universal sgRNA were designed for highly homologous sequences using MultiTargeter and visualised using Sequencher, creating unique sgRNA avoiding SNP and shared nucleotide locations targeting optimal edit sites.

**Conclusions:**

Using this framework, the approach has wider applications to all plant species regardless of ploidy number or highly homologous gene sequences.

**Significance statement:**

Using this framework, a best-practice approach to genome editing is possible in all plant species, including cannabis, delivering a comprehensive *in silico* evaluation of the cannabinoid pathway diversity from a large set of whole genome sequences. Identification of SNP variants across all genes could improve genome editing potentially leading to novel applications across multiple disciplines, including agriculture and medicine.

## Introduction

*Cannabis sativa* L. belongs to the *Cannabaceae* family and is one of the earliest domesticated plant species with archaeological evidence of cultivation beginning in China as early as 5000 B.C [1]. Cannabis has since been used throughout the world for its fibre in textiles, protein-rich seeds and therapeutic properties. The medicinal benefits of cannabis have been explored by many cultures around the world for centuries, with different preparations used to treat pain, inflammation and to improve appetite [2]. Today, cannabis is classed as an illicit drug in many countries, however, the consumption of cannabis for its psychoactive properties is estimated to be in excess of 190 million users worldwide [3].

Cannabis is an annual, wind pollinated herb, mainly dioceous but monoecious plants do exist. The number of species in the genus *Cannabis* is currently debated with reports suggesting a polytypic genus [4, 5] or as a monotypic, highly polymorphic species [6, 7]. The classification of cannabis has recently been suggested to follow its cannabinoid and terpene profile [8], however, three species of cannabis are generally accepted: *Cannabis sativa*, *Cannabis indica* and *Cannabis ruderalis* [7].

Cannabis contains a group of unique pharmacologically active chemical compounds called cannabinoids primarily produced in the glandular trichomes on female flowers. Phytocannabinoids represent a diverse group of C_21_ terpenophenolic compounds with a total of 120 cannabinoids currently reported [9].

The mammalian endocannabinoid system is comprised of endogenous cannabinoid receptors and metabolic enzymes that play a crucial role in homeostasis. The therapeutic potential for medicinal cannabis to aid in regulating physiological, immunological and behavioural conditions is of great interest. Reported *in vivo* effects in human and animal models indicate therapeutic applications in conditions such as multiple sclerosis [10], cancer [11], pain management [12] and epilepsy [13]. The highly polymorphic nature of cannabis is currently a limiting factor in reliable dosing quantities of cannabinoids, creating uncertainty in product efficiency.

Δ^9^-Tetrahydrocannabinol (THC), responsible for the psychoactive properties in cannabis, and Cannabidiol (CBD), non-psychoactive with diverse pharmacological properties, are the most abundant cannabinoids found in cannabis with their therapeutic properties being extensively reviewed [14]. Phytocannabinoids are synthesised in their acidic forms and undergo decarboxylation into their active neutral forms with heat or time [15, 16]. Due to the large variation of cannabis strains containing different levels of chemical variants, cannabinoid fractions are referred to as chemotypes. Initially, chemotypes of cannabis were classed as “drug-types” and “fibre-types” [17] representing THC+CBN/CBD quotient >1 or <1 respectively. It was later agreed that a plants chemotype was broken down into three major and two minor chemotypes as the current model [18]. Biosynthesis of the major cannabinoids, THC and CBD, from the common precursor cannabigerol (CBG) is performed by tetrahydrocannabinolic acid synthase (THCAS) and cannabidiolic acid synthase (CBDAS) [19, 20]. de Meijer *et*. *al* (2003) proposed the genetic determination for chemotypes as two alleles at a single gene locus, termed the B locus. The B_T_ allele encodes THCAS, and with the B_D_ allele encoding CBDAS. Those with high THC and low CBD have *B*_*T*_*/B*_*T*_ and *B*_*D*_*/B*_*D*_ genotypes respectively and contain high levels of CBD with little to no THC, and *B*_*T*_*/B*_*D*_ genotypes similar concentrations of THC and CBD. More recently, Grassa *et*. *al* (2018) completed the chromosome genome sequence assembly of cannabis finding that cannabinoid biosynthesis genes are not located at a single locus but are pericentromeric, nested in repeats leading to low levels of recombination.

**Fig 1:**
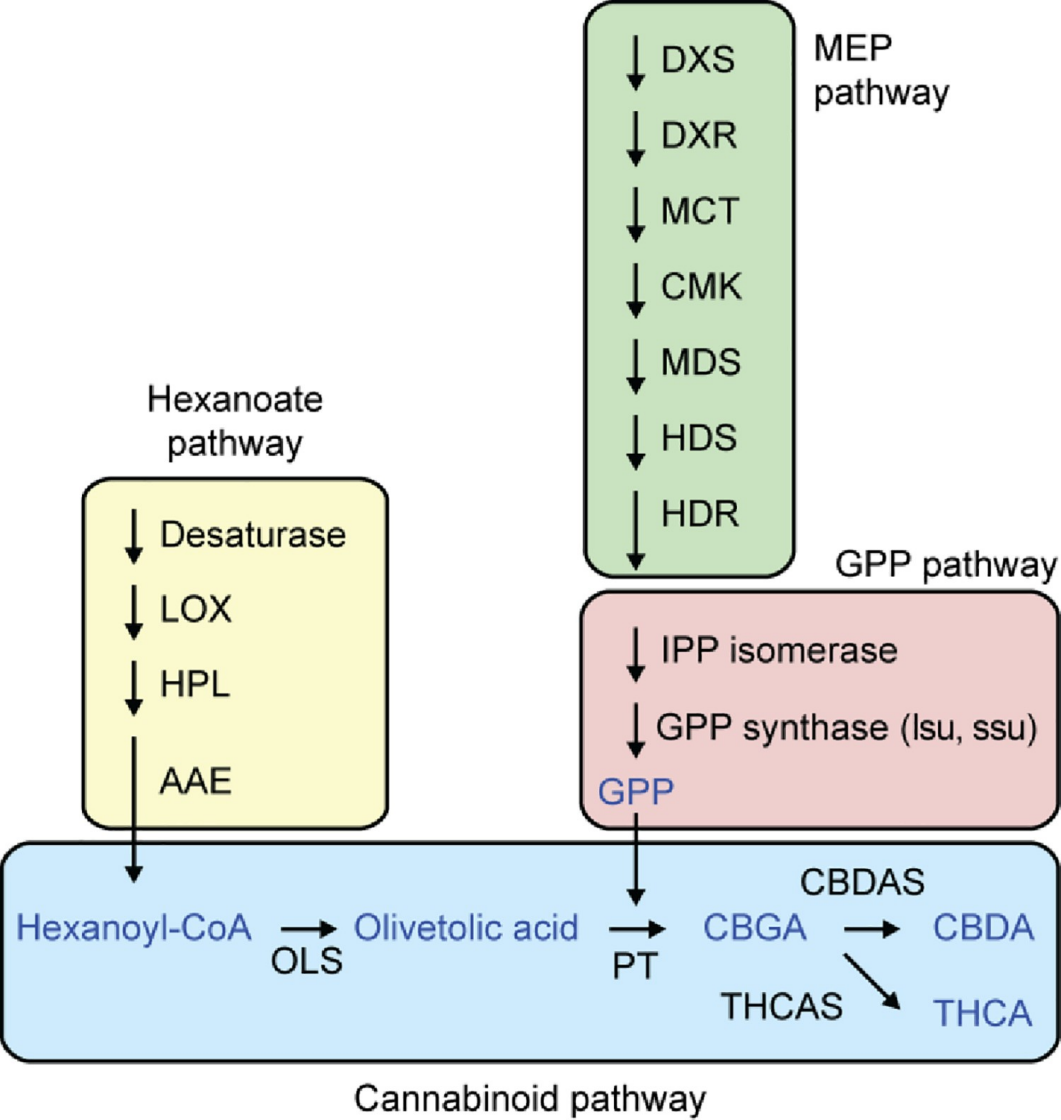
Overview of the cannabinoid biosynthesis pathways. Modified from van Bakel *et al*. (2011) [24].

Biosynthesis of cannabinoids is complex with numerous enzymatic steps and interactions. Fatty acids and isoprenoid precursors are synthesised via the hexanoate, methylerythritol 4-phosphate (MEP) and geranyl diphosphate (GPP) pathways. Hexanoyl-CoA is produced via the hexanoate pathway, acting as the substrate for olivetolic acid synthase (OLS) yielding OLA [21]. Prenyl sidechains are synthesised via the MEP pathway for the substrate for geranyl diphosphate synthesis. GPP and OLA are added by an aromatic prenyltransferase (PT) creating CBGA [22]. Finally, catalysation of THC and CBD oxidocyclases produce THCA and CBDA [23, 24] (Fig 1). Identification of all genes encoding biosynthetic enzymes now allows biotechnological approaches to control cannabinoid content by allowing genomically informed decisions on molecular breeding with tools such as genome editing.

The development of genome editing technologies, such as Clustered Regularly Interspaced Palindromic Repeats (CRISPR/Cas9), Zinc Finger Nucleases (ZFNs) and Transcription activator-like effector nucleases (TALENs) utilise sequence specific nucleases to induce a double strand break (DSB) at a specific genomic location through homologous binding of guide proteins [25]. Plants’ predominant repair pathway mechanism is through non-homologous end joining (NHEJ), and less often through homologous recombination (HR) [26]. NHEJ repairs the cut DNA without a homologous DNA template, however NHEJ can be error-prone, causing mutations such as base pair deletions, insertions or rearrangements [26, 27]. HR requires the provision of a DNA template, with homologous flanking regions used as a guide, to repair the break either correctly or by incorporating alterations that are desired into the DNA break point [28]. The use of genome editing techniques to manipulate gene function in a range of plant species has allowed for the generation of improved crop varieties, improved resistance and increased yield [29–31]. Within the CRISPR/Cas 9 system, the single-guide RNA (sgRNA), a 20nt oligo complementary to the gene of interest, guides the Cas9 endonuclease to the protospacer-adjacent motif (PAM) site, where Cas9 binds and cleaves the DNA strand [32]. Online tools available for sgRNA design and plasmid construction have been extensively reviewed [33] with CRISPR/Cas9 being broadly implemented in plants such as Arabidopsis, tobacco, rice and sorghum [34, 35]. ZFNs contain a tandem array of Cys2-Hys2 finger domains linked to the *Fok*I catalytic domain, with the finger domains each recognising 3bp of DNA [36]. The finger arrays are fused to the catalytic domain of *FokI* functioning as a dimer. Binding of the zinc-fingers to the target loci brings the two *FokI* monomers into close proximity causing them to dimerise, creating a DSB [37]. Similar in the mode of action to ZFNs, TALENs are comprised of a nonspecific *Fok*I nuclease domain fused to a DNA binding domain containing highly conserved repeats from the transcription activator-like effectors (TALEs) secreted by *Xanthomonas* spp. [38].

Off-target mutations caused by inefficient guide design and *FokI* monomer dimerisation could disrupt the functions of unintended genes, causing genetic instability and unintended cytotoxic effects. Single nucleotide polymorphisms (SNP) in genomic DNA across large, diverse populations will disrupt the homology-based binding of sgRNA, ZFs and TALEs with CRISPR/Cas9, ZFNs and TALENs. Target specificity is tightly controlled by sequence homology, with an increasing number of mismatches, off-target cleavage also increases [39]. Avoiding off-target effects is critically important for effective and efficient genome editing, with the need for genomically informed designs based on thorough deep-read genome sequencing being more important than ever. If these tools are to be regulated and used in product design, absolute confidence in design based on homology is needed.

In this study we outline the best practice workflow for identifying target sequences and their corresponding design using sgRNA in cannabis for the manipulation of the entire pathway of THC and CBD synthesis. Through genomically informed decisions based on previously published cannabis pangenome, generic and specific sgRNA can be designed using online tools to successfully target genes of interest with no *in silico* detected off-targets. The workflow here can help make informed decisions on gene targeting in cannabis, leading to novel cannabinoid production by targeting cannabis biosynthesis genes, accelerating the understanding of the relationships of genes in cannabinoid production.

## Materials and methods

### Genome sequence assembly

Genome sequence assembly of the Cannbio-2 genotype were performed by Braich *et al*. [40] (https://doi.org/10.46471/gigabyte.10) with a brief summary given here. RaGOO [41] was used to scaffold the draft genome of Cannbio-2 to chromosome scale pseudomolecules with CBDRx genome assembly as the reference. Access to CBDrx genome (known as cs10 in NCBI) is available through The European Nucleotide Archive (PRJEB29284) (https://www.ebi.ac.uk/ena/data/view/PRJEB29284). PK and Finola genome assemblies were accessed through the NCBI BioProject database (PRJNA73819) (https://www.ncbi.nlm.nih.gov/bioproject/?term=PRJNA73819).

### Cannbio-2 and pangenome gene analysis

Cannabinoid biosynthesis genes were accessed from a variety of sources and public databases (Table 1) to annotate Cannbio-2. Sequences were downloaded and used as a query for BLAST analysis against the Cannbio-2 genome assembly with an e-value threshold set at <10^−10^. Identified regions of interest from the reference genome were annotated using NCBI nBLAST to confirm sequence identity and MEGANTE [42] and coding sequences (cds) visualised using FGENESH [43]. Sequences are available in S1 Table in S1 Data.

**Table 1:** Source of gene query/NCBI accession number and gene copy and homolog number for available genomes discovered using BLAST.

Gene		Pathway	Cannbio-2	CBDrx	Finola	PK V2
	NCBI Accession Number/Source of Query		Copy number/homologs			
DXS1	KY014576.1	MEP	1	1	-	1
DXS2	KY014577.1	MEP	1	1	-	1
DXR	KY014568	MEP	1	1	1	2
MCT	KY014578	MEP	1	1	1	1
CMK	KY014575	MEP	1	1	1	1
MDS	HQ734721.1	MEP	1	1	1	1
HDS	KY014570.1	MEP	1	1	1	1
HDR	KY014579.1	MEP	1	1	1	1
IPP/IPI	KY014569.1	GPP	1	-	1	1
GPP LSU	KY014573.1	GPP	1	1	1	1
GPP SSU	KY014567.1	GPP	1	1	-	1
FAD2	PK genome, scaffold71447:2,827–3,852	Hexanoate	4	5	7	3
LOX	PK genome, scaffold53609:3,286–7,284	Hexanoate	1	1	1	1
HPL	PK genome, scaffold14797:30,184–30,623	Hexanoate	1	1	1	1
AAE1	JN717233	Hexanoate	1	1	-	1
OLS	EU551162.1	Cannabinoid	1	1	1	2
OAC	JN679224.1	Cannabinoid	2	1	1	2
GOT	Publication number: US20120144523A1	Cannabinoid	1	1	1	1
CBDAS	AB292682	Cannabinoid	9^1^	11 total	9 total	14 total
THCAS	AB057805	Cannabinoid	1			
CBCAS	Publication number: WO/2015/196275	Cannabinoid	3^2^			

^1^2 genes and 7 homologs.

^2^2 genes and 1 homolog.

Publicly available cannabis genomes were downloaded (as described above) and were BLAST analysed using Cannbio-2 gene sequences described here with an e-value threshold set at <10^−10^ to determine copy numbers within each respective genome (Table 1).

### SNP discovery

SNP discovery was performed by Braich *et al*. [44], with a brief summary given here. Genomic DNA was extracted from fresh leaf material from a range of 660 mixed cultivars (high CBD, high THC, balanced THC:CBD, male and female plants) using DNeasy 96 Plant Kit (QIAGEN, Hilden, Germany) according to the manufacturer’s instructions. Each library was prepared using enzymatic shearing using MspJI (NEB, MA, USA) in-house library prep protocol and sequenced on a HiSeq3000 instrument (Illumina Inc. San Diego, CA, USA)The resulting sequence data was reference aligned to the Cannbio-2 genome assembly previously described, using the BWA MEM algorithm [45]. Variants were identified using SAMtools [46] and a bed file with scaffold regions of interest matching to gene sequences of cannabinoid biosynthesis genes was created. Alignments were sorted and used for variant calling with an adjusted mapping quality (-C 50) and minimum read depth of 5 generating a consensus sequence. Consensus sequences for CDS sequences of genes of interest are available in S2 Table in S1 Data.

### Determination between allele or gene

Presence of an allele, or extra copies of a gene, were determined based on genomic nucleotide multiple sequence alignments using MUSCLE [47]. Sequences of similar length with alignment similarity between 80–98%, which produced identical translated proteins were determined as alleles. Where large variation existed between genomic nucleotide sequence length or content, or where nucleotide sequences were <1000bp, predicted mRNA sequences were used from FGENESH [44] for alignment. Alleles were determined if similarity equalled >98%. Additional gene copies were determined if greater than two haplotypes were found with similarities >90% but <98%, due to cannabis being an outbreeding species and the Cannbio-2 genome sequence assembly is based off a heterozygous plant.

### sgRNA design and confirmation

CHOPCHOP [48], CRISPR MultiTargeter [49], Crispor [50] and ZiFit [51] were used for the selection of sgRNAs for use with CRISPR-Cas9. Entire CDS region, calculated by FGENESH [43] and MEGANTE [42], were used as search queries. sgRNA on and off-target parameters suggested by each online tool was used. For visual confirmation of SNP avoidance, sgRNAs were manually aligned to Cannbio-2 and consensus sequences using Sequencher [52]. sgRNA designs are available in S3 Table in S1 Data.

## Results

### Genome mining for cannabinoid biosynthesis genes

To locate all the genes involved in cannabinoid biosynthesis, query references were downloaded from publicly available databases (Table 1) and BLAST analyses was performed against the Cannbio-2 genome assembly.

All genes in the MEP, GPP, Hexanoate and Cannabinoid pathway were identified (Table 1). Two 1-deoxy-D-xylulose 6-phosphate synthase (DXS) genes were discovered in the MEP pathway alongside single copies of 1-deoxy-D-xylulose 5-phosphate reductoisomerase (DXR), 4-diphosphocytidyl-2C-methyl-D-erythritol synthase (MCT), 4-diphosphocytidyl-2-C-methyl-D-erythritol kinase (CMK), 2C-methyl-D-erythritol 2,4-cyclodiphosphate synthase (MDS), 4-hydroxy-3-methylbut-2-en-1-yl diphosphate synthase (HDS) and 1-hydroxy-2-methyl-2-(E)-butenyl 4-diphosphate reductase (HDR). Single genes of isopentenyl diphosphate isomerase (IPP/IPI), geranyl pyrophosphate synthase (GPP), small and large subunits, were identified in the GPP pathway. In the hexanoate pathway, four copies of fatty-acid desaturase (FAD2) were identified using the Purple Kush (PK) desaturase gene sequence as the query. Translated proteins from all FAD2 homologs were tBLASTn analysed for confirmation of correct annotation and all are believed to be involved in cannabinoid biosynthesis. Lipoxygenase (LOX) and hydroperoxide lyase (HPL) were identified using the associated PK gene sequences as the queries with very low (<1%) sequence variation existing. Acyl-activating enzyme (AAE1) was found using previously published sequences (Table 1) amongst the AAE superfamily, containing 15 AAE homologs. Translated AAE1 annotation was confirmed using tBLASTn and isolated from the large superfamily of highly homologous gene sequences. In the cannabinoid pathway a single copy of olivetol synthase (OLS) was discovered with >98% identity to deposited OLS sequences in NCBI. Two copies of olivetolic acid cyclase (OAC) were discovered. The CDS of the set of alleles and a single copy of OAC were aligned and 14 SNPs exist between the set. All OAC sequences were correctly annotated using MEGANTE and tBLASTn to confirm copy number. Two complete identical, functional CBDAS-like genes were discovered (CBDAS-like#1 and #2) with three closely related homologs also existing (CBDAS-like#3–5). CBDAS-like homologs contain several SNPs causing sequence variation in translated protein sequences. Four truncated CBDAS homologs were also discovered (CBDAS-truncated#1–4), with each containing stop codons resulting in truncated protein sequences. Two complete copies of cannabichromenic acid synthase (CBCAS) were found (CBCAS#1+#2) with identical sequences except at base pair 662 with a SNP of C to T, though identical proteins are predicted. One closely related truncated homolog of CBCAS was also discovered (CBCAS-truncated) producing a substantially shorter predicted protein sequence. One single copy of THCAS was also discovered.

### Pan-genome copy number variance comparison

Within the publicly available cannabis genome sequences, the assembled gene set was then used to query gene copy number and identify potential homologs. Differences exist between the datasets in terms of gene copy number due to the resolution of the sequence data, genetic mapping, scaffolding technologies and natural variation in different genomes. Variations in gene presence and copy number, using the assembled reference gene list, exist for DXS1, DXS2, DXR, IPP/IPI, GPP_SSU, FAD2, AAE1, OLS, OAC, CBDAS, THCAS and CBCAS (Table 1). Within the Finola genome, DXS1, DXS2, GPP_SSU and AAE1 were not discovered, with copy number variation existing for FAD2, OLS and OAC when compared to Cannbio-2 (Table 1). Within the CBDrx genome, no copy of IPP/IPI was discovered, which is confirmed by the most recent release of the CBDRx genome. Copy number variations exist for FAD2 compared to Cannbio-2, with 4 FAD2 genes being discovered in Cb-2 and 5 in CBDRx. The updated PK genome had at least one copy of each gene, with variations in copy number existing for DXR, FAD2, OLS and OAC and synthase genes compared to Cannbio-2.

### Analysis of SNPs and informed sgRNA design

**Fig 2:**
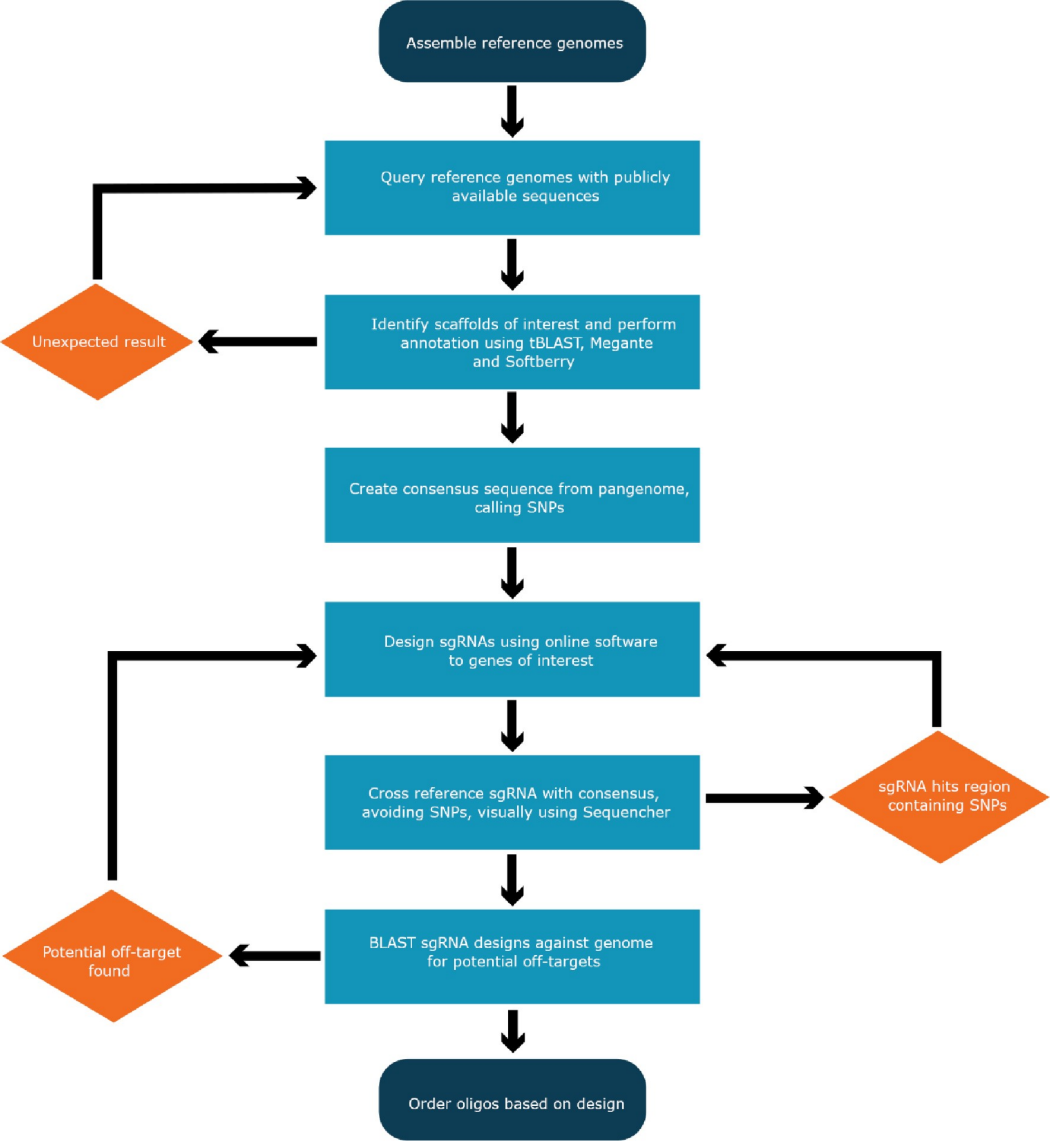
Informed genome editing pipeline for intelligent design of sgRNA.

To assess gene variation, the six hundred and sixty whole genomes that were sequenced were used to establish a resource of SNP locations (consensus sequence) (S3 Table in S1 Data), which were then overlayed onto the identified genes integral to the cannabinoid biosynthesis. With the exception of FAD2, which belongs to a large, diverse family of desaturases, the cannabinoid biosynthesis genes are highly conserved with little variation within their sequences (Table 2). Each consensus sequence containing SNP locations was then used for intelligent guide designs to avoid all known nucleotide variations, creating universal sgRNA which can be broadly used on any cannabis genotype, and in the instance of highly similar gene sequences, unique sgRNA designed to target only a specific gene of interest (Fig 2). Sequences from the reference genome were entered into the online design tools CHOPCHOP, CRISPR MultiTargeter, Crispor and ZiFit to generate sgRNA based on their preferred scoring matrixes followed by manual and visual comparison. Taking the highest-ranking scores from each online tool, which predict off-targeting potential and greatest binding affinity, each sgRNA was visualised, using Sequencer, to identify regions the sgRNA would target, whether that be in regions of sequence homology across the pan-genome or in regions consisting of SNPs. A total of 145 sgRNAs were designed targeting every gene in the combined pathways (S1 Table in S1 Data). The sgRNA generated consists largely of a pool of universal sequences, which regardless of cultivar used, can target each gene in the combined pathways through the use of the consensus sequence generated. Multiple Cannbio-2 specific sgRNA were also designed in regions where sequence heterogeneity towards the 5’ translated regions dictated universal sgRNA design was not possible. All sgRNA were re-BLAST analysed against the reference genome for detection of off-site targeting, with results confirming no complete 20-nt sgRNA had potential off targets outside their respective gene sets.

**Table 2:** Gene length defined as genome base-pair length including introns, and location within Cannbio-2 with accompanying consensus SNP data from pangenome.

Gene	Gene Length	CB-2 Genome Location	# SNPs in Pangenome
DXS1	3601	Chr:9 14103141–14103512	6
DXS2	2892	Chr:4 79846660–79849546	71
DXR	3689	Chr:3 10253558–10257868	68
MCT	4242	Chr:4 36521598–36525845	155
CMK	4031	Chr:2 12810228–12814256	103
MDS	1946	Chr:5 86405983–86407926	70
HDS	5383	Chr:2 100426265–100431627	211
HDR	2309	Chr:X 7955658–7957964	76
IPP/IPI	2921	Chr:2 13601913–13604831	50
GPP_LSU	1281	Chr:4 91310699–91311977	31
GPP_SSU	1061	Chr:6 55780334–55781392	19
FAD2#1	1123	Chr:2 104383871–104384992	57
FAD2#2	1085	Chr:2 104394699–104395781	52
FAD2#3	1091	Chr:2 104401152–104402226	53
FAD2#4	1084	Chr:2 104420931–104422048	25
LOX	4162	Chr:2 102127730–102131887	133
HPL	7201	Chr:8 53062338–53070863	200
AAE1	6688	Chr:3 50354410–50361096	220
OLS	1418	Chr:8 61667472–61668887	35
OAC	692^1^	Chr:9 5793422–5794110	17
OAC#2	548^1^	Chr:9 6925677–6926172	15
GOT	7350	Chr:X 65676960–65684340	264
THCAS	1868	Chr:7 29533343–29535211	37
CBCAS#1	1635	Chr:7 29465648–29467283	2
CBCAS#2	1635	Chr:7 29577848–29579483	2
CBCAS-truncated	1420	Chr:7 29518627–29519784	5
CBDAS-like#1	1900	Chr:7 33131612–33133245	3
CBDAS-like#2	1628	Chr:7 33199940–33201573	0
CBDAS-like#3	1700	Chr:7 33234459–33236068	12
CBDAS-like#4	1500	Chr:7 33275773–33277406	24
CBDAS-like#5	1704	Chr:7 33371944–33373577	13
CBDAS-truncated#1	449	Chr:7 33122514–33123491	9
CBDAS-truncated#2	1839	Chr:7 33341204–33342916	14
CBDAS-truncated#3	990	Chr:7 34564480–34566132	46
CBDAS-truncated#4	1113	Chr:7 34569433–34570813	40

^1^complete CDS only.

## Discussion

Phytocannabinoids are of particular interest for their pharmacological applications in a growing number of medical conditions. Knowledge and understanding of the gene interactions and their relationship to final cannabinoid concentration can facilitate improved cannabis strains with desired novel cannabinoid levels. Creating a pangenome consensus of each gene in the contributing pathways allows for genomically informed decisions, based on known SNP location and frequency as well as presence absence variations (PAV), for crop improvement by means of genome editing. Using publicly available sequence information, at least one full length transcript for all genes involved in cannabinoid biosynthesis were found agreeing with previous genome sequencing and genome mining reports [24, 53]. Gene copy number in the MEP pathway also agrees with previously published analysis [53]. Two DXS genes were discovered, with previous reports showing DXS1 having elevated expression levels in photosynthetic tissues, underlining its importance in isoprenoid production [54]. DXS2 accumulates in the roots with expression patterns suggesting synthesis of specific isoprenoids, however, it’s role in cannabinoid biosynthesis is yet to be determined. Multiple genes for DXR [55], HDR [56] and IPI/IPP [57] have been previously reported, however, only singular copies of these genes were discovered in the Cannbio-2 genome. It is possible that multiple copies of these genes could be responsible for the accumulation of cannabinoid precursors, leading to novel cannabinoid levels. Fatty acid desaturase enzymes belong to two large multifunctional classes, either membrane bound, or soluble. The desaturase of interest in cannabinoid production, FAD2, is involved in the hexanoate pathway, leading to the production of hexanoyl-CoA, the first precursor in the cannabinoid pathway. Despite the complexity of the number of FAD2 gene sequences, it is believed that the correct version was identified, although our data shows four copies of this gene, where previous comparative studies discovered seven gene copies in the Finola genome [58] and only 2 copies in the CBDRx genome [59]. Further evidence of gene copy number variance, across published genomes, exists for OLS, and OAC posing the question if gene copy number directly influences chemovar determination. Previous studies have utilised short read sequence data in the identification of gene sequences and due to the anticipated degree of sequence similarity from the duplicate gene copies, taking a reference-aligning approach would be inaccurate to use the data generated to infer CNVs. However, with the availability of long read sequencing technology that can generate sequence data through extended repetitive regions, describing genome architecture and gene sequence and structure at a much higher level, makes it a reliable platform to use for the determination of CNVs. THC-rich PK cultivar has two copies of OLS and OAC, whereas CBD-rich cultivar, CBDrx, has just one copy of each from our BLAST search results, though 2 copies of OLS and no copies of OAC are reported. The presence of OAC is a polyketide synthase enzyme catalyses olivetolic acid, which forms the polyketide nucleus of cannabinoids [21]. This suggests that this particular polyketide was not included in the CBDRx genome, though it is considered essential for cannabinoid biosynthesis. The Cannbio-2 cultivar, with relatively equal (1.8:1) THC and CBD cannabinoid concentrations contains a single copy of OLS and 2 copies of OAC.

The exact relationship between gene copy number and cannabinoid production needs to be further studied through metabolic engineering in heterologous hosts or through genome editing. Using the discovered synthase genes from the Cannbio-2 genome sequence as the query against CBDrx, Finola and PK genomes, the total number of synthase genes varies considerably between the cultivars. In the CBDrx genome [59] 16 synthase genes are reported, however only 11 were discovered in CBDrx using sequences from Cannbio-2 as queries. Identification of which synthase genes were not identified is difficult due to the nested repeating nature of synthase genes around the centromere.

As long read sequencing is error prone, the correct assembly of *CBDAS* in the Cannbio-2 assembly has proven problematic, potentially exacerbated due to the hybrid nature of the genotype. It is therefore likely that the CBDAS gene has been incorrectly assembled and either a chimeric version of the functional and non-functional gene alleles, or that the non-functional allele only has been assembled, most likely as the gene that is referred to as CBDAS-truncated#3. The Cannbio-2 genome clearly has a functional CBDAS allele as a 100% identity sequence has been identified from the transcriptome data set [60] (Cannbio_016865).

Grassa *et*.*al* (2021) has identified the total number of potential synthase genes in reference to a sequence alignment to *THCAS* mRNA >82%. The variation in synthase genes is most likely due to PAV across different cultivars, which in the case of maize is common [61]. Total synthase gene number for Finola and PK is not given in the original genome [62], however 9 and 14 genes were found when querying with Cannbio-2 sequences. Grassa *et*.*al* (2021) has identified 5 and 16 synthase genes within the PK and Finola from their respective approach to discovering copy numbers.

THCAS and CBDAS CNV have recently been reported from multiple cannabis cultivars with similar findings that this CNV partially explains variation in cannabinoid content [63, 64]. Multiple gene copies is a known method to increase production of secondary metabolites [65] which could lead to the understanding that increased copy number of synthase genes would in turn increase cannabinoid production. However, possibly a greater explanation of increased cannabinoid potency was discussed by Grassa *et al*. (2018) with the discovery that separate QTLs, not linked to synthase gene clusters, were responsible for up to 17% variation in cannabinoid quantity. This could possibly help explain the current gene copy number variation in the observed genes mentioned.

Complete absence of sequence data is present for specific genes in the CBDrx, Finola and PK genomes posing the question whether genome assembly, or actual PAV mechanisms are responsible. Within the Finola genome, 4 genes could not be identified. Both forms of DXS are not present and with previous studies demonstrating DXS knock down lines produce reduced levels of isoprenoids and contain more severe phenotypic characterisations [66, 67], suggesting the fragmented genome failed to identify and assemble the specific genes of interest. GPP SSU and AAE1 were also not identified, however, from previous reports both these genes are critical for isoprenoid and cannabinoid production indicating they are missed in the genome assembly. AAE1 was found to be the gene which synthesises hexanoyl-CoA from hexanoate supplying the cannabinoid pathway [68] and since Finola still produces cannabinoids, it is concluded that it was also an assembly error. GPP is a heterodimer requiring both subunits, large and small, for optimum activity. GPP activity has shown to still be active but at lower levels when the small subunit was inactive [69], however both subunits were still present, suggesting the absence of GPP SSU in the Finola genome is also due to assembly error. The absence of IPP/IPI in the CBDrx genome is also strongly suggested to be due to assembly error, since previous studies on *Arabidopsis* double mutant knockdown of IPP/IPI produced dwarfism and male sterility [70].

The SNP location resource revealed some genes are more highly conserved than others. The variable conservative nature of genes was observed indicating a continuing evolution of recombination and divergence. Comparative analysis of SNPs present in genes of variable copy number in Cannbio-2, CBDrx, Finola and PK genomes was performed (excluding results of no gene presence). Through multiple sequence alignments of coding sequences, it was observed that the presence of SNP’s occurred in the extra gene copy where the presence of homozygous alleles exists. This suggests that either sequencing error has occurred, or in fact there is an extra copy of the gene and a set of alleles. Within the Cannbio-2 genome, OAC produced three sequence similarity matches with two sequences determined as alleles with an extra copy of the gene existing as a truncated version of the gene. When gene sequences were aligned, SNPs occurred in all genes and when translated, nearly identical protein sequences (>99%) were produced confirming that an extra copy of the gene was present, potentially in a hemizygous condition. Within the PK genome, copy number variation exists for OLS and OAC. In a similar way to OAC in the Cannbio-2 genome, OLS produced three hits, two of which were determined to be alleles and one to be an extra copy. SNPs existed in all three sequences when coding regions were aligned with similar results obtained from protein sequence alignment. Initial alignment of both OAC hits, in PK, found a 98.5% similarity in genomic sequences, however no gene prediction was possible on one of the sequences, possibly due to a premature stop codon from a SNP rendering this gene inactive potentially indicating that it exists as a pseudogene.

How this copy number variation contributes to differential cannabinoid production is yet to be fully elucidated, however using the known SNP location for each extra copy gene in Cannbio-2, sgRNA could be designed to help understand this relationship. Using multiple online tools for the design of sgRNA ensured that all possible guide designs could be assessed for *in silico* off-targeting. Each tool implements different scoring rules based on off-targets, mismatches, efficiency score, existence of self-complimentary regions, GC content, location of guide and multiple sequence alignments [48, 49]. Due to the diversity in gene content and sequence variation and the absence of a well characterised pan-genome for cannabis, analysis by these multiple tools was necessary and essential. The presence of a PAM site is necessary for sgRNA binding and even though these tools scanned the gene sequence for the PAM sites, results occasionally varied between the online tools. Visualisation of sgRNAs was clear using CHOPCHOP compared to the other tools and regularly provided the best guide designs. However, when highly homologous sequences were used MultiTargeter was able to perform sequence alignments and produce unique sgRNA for each sequence, a feature not possible within the other tools. Designing the sgRNA for the unique synthases were first run using MultiTargeter and further verified using CHOPCHOP for visualisation. sgRNA designed were targeted to the earliest possible exon for maximum likelihood of a frame shift mutation. The error prone nature of NHEJ often occurs with small deletions, or insertions, occurring at the DSB leading to protein misfolding and thus production of a knockout gene. Each identified gene, with accompanying allele where applicable, were analysed and sgRNAs were designed to be either universal, inactivating both related genes, or if sequence heterozygosity exists, specific sgRNA were designed (S1 Table in S1 Data). Mutational studies identifying differential expression in isoprenoid biosynthesis genes, including DXS [67], DXR [71], IPP/IPI [70] and MDS [72] have previously been reported. Mutational studies on the unique synthase genes are yet to be reported, potentially due to the high homology between enzymes. Using genome editing, sequence homogeneity between synthase genes could potentially lead to off-target editing, with targets suggested to have at least several nucleotides different for discrimination [73]. Where possible, each synthase gene, and accompanying homologs, had universal and specific sgRNA designed that could be used regardless of cultivar, strain or population chosen as the target. The reported sequence similarity between THCAS, CBDAS and CBCAS, up to 95% [62], requires precise, intelligent design, using multiple online tools and a large consensus population to improve the likelihood of correct gene knock down. Potential off targeting predictions given by sgRNA online tools currently use the previously fragmented genome of PK [24]. To circumvent this, each sgRNA was used as a query to BLAST against the Cannbio-2 genome for potential off-targets. From the BLAST results no sgRNA had an unexpected sequence match elsewhere in the genome, however singular nucleotide mismatches do occur. How these mismatches are tolerated during directed genome editing is yet to be determined, however it is expected that off-targeting will be more prevalent with more highly homologous gene sets.

Applying this logical workflow *in silico* is the benchmark standard, essential to ensure that correct genes and associated SNPs are identified before genome editing can begin. This approach has wider applications in all genome editing efforts within species that have paleo-polyploidy, large PAV gene populations or crop species with high levels of variations within the genome. This workflow explains each step taken and the tools to use to obtain universal or specific sgRNA to any gene of choice quickly and effectively, where each step can encounter issues and how to correct them making this approach critical for effective genome editing with minimal off-targeting. This same approach can easily be applied to the more recent CRISPR-Cas12a system which has been gaining popularity with editing plant genomes. The availability of fully sequenced genomes, pangenomes and the ability to accurately predict potential off-target effects and edits makes this method applicable to all plant gene editing applications regardless of species. Only recently the ability to analyse the cannabis genome has become available showing that using this approach, with current technologies available, this method can be used quickly and effectively. Even with the limited literature and resources available for completed cannabis genomes, quick, intelligent design for genome editing in cannabis is now possible. Understanding the effect of gene copy number, PAV and SNP location and density on cannabinoid production can help create unique cannabinoid profiles for medicinal purposes.

## Supporting information

S1 Data (XLSX)

## References

[1] Li H-L. An archaeological and historical account of cannabis in China. Econ Bot. 1973;28(4):437–48.

[2] Kalant H. Medicinal use of cannabis: history and current status. Pain Res Manag. 2001;6(2):80–91. doi: 10.1155/2001/469629 11854770

[3] Office on Drugs and Crime UN. World Drug Report 2017. Executive Summary—Conclusions and Policy Implications. 2017 [cited 2018 Jun 25]; Available from: https://www.unodc.org/wdr2017/field/Booklet_1_EXSUM.pdf

[4] Emboden WA. Cannabis—a polytypic genus. Econ Bot. 1974;28(3):304.

[5] Hillig KW. Genetic evidence for speciation in Cannabis (Cannabaceae). Genet Resour Crop Evol. 2005;52(2):161–80.

[6] Doorenbos NJ, Fetterman PS, Quimby MW, Turner CE. Cultivation, extraction, and analysis of Cannabis sativa L. Ann N Y Acad Sci. 1971;191(1):3–14.

[7] Small E, Cronquist A. A practical and natural taxonomy for Cannabis. Taxon. 1976;405–35.

[8] Piomelli D, Russo EB. The Cannabis sativa versus Cannabis indica debate: an interview with Ethan Russo, MD. Cannabis cannabinoid Res. 2016;1(1):44–6. doi: 10.1089/can.2015.29003.ebr 28861479 PMC5576603

[9] ElSohly MA, Radwan MM, Gul W, Chandra S, Galal A. Phytochemistry of Cannabis sativa L. In: Phytocannabinoids. Springer; 2017. p. 1–36. 10.1007/978-3-319-45541-9_1 28120229

[10] Alexander SPH. Therapeutic potential of cannabis-related drugs. Prog Neuro-Psychopharmacology Biol Psychiatry. 2016;64:157–66. doi: 10.1016/j.pnpbp.2015.07.001 26216862

[11] Ramer R, Hinz B. Inhibition of cancer cell invasion by cannabinoids via increased expression of tissue inhibitor of matrix metalloproteinases-1. JNCI J Natl Cancer Inst. 2008;100(1):59–69. doi: 10.1093/jnci/djm268 18159069

[12] Perez J, Ribera MV. Managing neuropathic pain with Sativex®: a review of its pros and cons. Expert Opin Pharmacother. 2008;9(7):1189–95. doi: 10.1517/14656566.9.7.1189 18422475

[13] Devinsky O, Marsh E, Friedman D, Thiele E, Laux L, Sullivan J, et al. Cannabidiol in patients with treatment-resistant epilepsy: an open-label interventional trial. Lancet Neurol. 2016;15(3):270–8. doi: 10.1016/S1474-4422(15)00379-8 26724101

[14] Cascio MG, Pertwee RG, Marini P. The Pharmacology and Therapeutic Potential of Plant Cannabinoids. In: Cannabis sativa L-Botany and Biotechnology. Springer; 2017. p. 207–25.

[15] Flores-Sanchez IJ, Verpoorte R. Secondary metabolism in cannabis. Phytochem Rev. 2008;7(3):615–39. doi: 10.1093/pcp/pcn150 18854334

[16] Trofin IG, Dabija G, Vaireanu D, Filipescu L. Long-term storage and cannabis oil stability. Rev Chim. 2012;63(3):293–7.

[17] Fetterman PS, Keith ES, Waller CW, Guerrero O, Doorenbos NJ, Quimby MW. Mississippi‐grown cannabis sativa L.: Preliminary observation on chemical definition of phenotype and variations in tetrahydrocannabinol content versus age, sex, and plant part. J Pharm Sci. 1971;60(8):1246–9. doi: 10.1002/jps.2600600832 5127101

[18] Pacifico D, Miselli F, Micheler M, Carboni A, Ranalli P, Mandolino G. Genetics and Marker-assisted Selection of the Chemotype in Cannabis sativa L. Mol Breed. 2006;17(3):257–68.

[19] Taura F, Morimoto S, Shoyama Y, Mechoulam R. First direct evidence for the mechanism of. DELTA. 1-tetrahydrocannabinolic acid biosynthesis. J Am Chem Soc. 1995;117(38):9766–7.

[20] Taura F, Morimoto S, Shoyama Y. Purification and characterization of cannabidiolic-acid synthase from Cannabis sativa L. Biochemical analysis of a novel enzyme that catalyzes the oxidocyclization of cannabigerolic acid to cannabidiolic acid. J Biol Chem. 1996;271(29):17411–6. doi: 10.1074/jbc.271.29.17411 8663284

[21] Gagne SJ, Stout JM, Liu E, Boubakir Z, Clark SM, Page JE. Identification of olivetolic acid cyclase from Cannabis sativa reveals a unique catalytic route to plant polyketides. Proc Natl Acad Sci. 2012;109(31):12811–6. doi: 10.1073/pnas.1200330109 22802619 PMC3411943

[22] Taura F, Sirikantaramas S, Shoyama Y, Yoshikai K, Shoyama Y, Morimoto S. Cannabidiolic‐acid synthase, the chemotype‐determining enzyme in the fiber‐type Cannabis sativa. FEBS Lett. 2007;581(16):2929–34. doi: 10.1016/j.febslet.2007.05.043 17544411

[23] Fellermeier M, Eisenreich W, Bacher A, Zenk MH. Biosynthesis of cannabinoids: Incorporation experiments with 13C‐labeled glucoses. Eur J Biochem. 2001;268(6):1596–604. doi: 10.1046/j.1432-1033.2001.02030.x 11248677

[24] Van Bakel H, Stout JM, Cote AG, Tallon CM, Sharpe AG, Hughes TR, et al. The draft genome and transcriptome of Cannabis sativa. Genome Biol. 2011;12(10):R102. doi: 10.1186/gb-2011-12-10-r102 22014239 PMC3359589

[25] Voytas DF. Plant genome engineering with sequence-specific nucleases. Annu Rev Plant Biol. 2013;64. doi: 10.1146/annurev-arplant-042811-105552 23451779

[26] Gorbunova V, Levy AA. Non-homologous DNA end joining in plant cells is associated with deletions and filler DNA insertions. Nucleic Acids Res. 1997;25(22):4650–7. doi: 10.1093/nar/25.22.4650 9358178 PMC147090

[27] Van Gent DC, Van Der Burg M. Non-homologous end-joining, a sticky affair. Oncogene. 2007;26(56):7731. doi: 10.1038/sj.onc.1210871 18066085

[28] Siebert R, Puchta H. Efficient repair of genomic double-strand breaks by homologous recombination between directly repeated sequences in the plant genome. Plant Cell. 2002;14(5):1121–31. doi: 10.1105/tpc.001727 12034901 PMC150611

[29] Kumar S, AlAbed D, Worden A, Novak S, Wu H, Ausmus C, et al. A modular gene targeting system for sequential transgene stacking in plants. J Biotechnol. 2015;207:12–20. doi: 10.1016/j.jbiotec.2015.04.006 25913173

[30] Ainley WM, Sastry‐Dent L, Welter ME, Murray MG, Zeitler B, Amora R, et al. Trait stacking via targeted genome editing. Plant Biotechnol J. 2013;11(9):1126–34. doi: 10.1111/pbi.12107 23953646

[31] Shukla VK, Doyon Y, Miller JC, DeKelver RC, Moehle EA, Worden SE, et al. Precise genome modification in the crop species Zea mays using zinc-finger nucleases. Nature. 2009;459(7245):437. doi: 10.1038/nature07992 19404259

[32] Mali P, Yang L, Esvelt KM, Aach J, Guell M, DiCarlo JE, et al. RNA-guided human genome engineering via Cas9. Science (80-). 2013;339(6121):823–6. doi: 10.1126/science.1232033 23287722 PMC3712628

[33] Cui Y, Xu J, Cheng M, Liao X, Peng S. Review of CRISPR/Cas9 sgRNA Design Tools. Interdiscip Sci Comput Life Sci. 2018;1–11. doi: 10.1007/s12539-018-0298-z 29644494

[34] Belhaj K, Chaparro-Garcia A, Kamoun S, Nekrasov V. Plant genome editing made easy: targeted mutagenesis in model and crop plants using the CRISPR/Cas system. Plant Methods. 2013;9(1):39. doi: 10.1186/1746-4811-9-39 24112467 PMC3852272

[35] Jiang W, Zhou H, Bi H, Fromm M, Yang B, Weeks DP. Demonstration of CRISPR/Cas9/sgRNA-mediated targeted gene modification in Arabidopsis, tobacco, sorghum and rice. Nucleic Acids Res. 2013;41(20):e188–e188. doi: 10.1093/nar/gkt780 23999092 PMC3814374

[36] Wolfe SA, Nekludova L, Pabo CO. DNA recognition by Cys2His2 zinc finger proteins. Annu Rev Biophys Biomol Struct. 2000;29(1):183–212. doi: 10.1146/annurev.biophys.29.1.183 10940247

[37] Kim Y-G, Cha J, Chandrasegaran S. Hybrid restriction enzymes: zinc finger fusions to Fok I cleavage domain. Proc Natl Acad Sci. 1996;93(3):1156–60. doi: 10.1073/pnas.93.3.1156 8577732 PMC40048

[38] Joung JK, Sander JD. TALENs: a widely applicable technology for targeted genome editing. Nat Rev Mol cell Biol. 2013;14(1):49. doi: 10.1038/nrm3486 23169466 PMC3547402

[39] Fu Y, Foden JA, Khayter C, Maeder ML, Reyon D, Joung JK, et al. High-frequency off-target mutagenesis induced by CRISPR-Cas nucleases in human cells. Nat Biotechnol. 2013;31(9):822. doi: 10.1038/nbt.2623 23792628 PMC3773023

[40] Braich S, Baillie RC, Spangenberg GC, Cogan NOI. A New and Improved Genome Sequence of Cannabis sativa. Gigabyte. 2020;1. 10.46471/gigabyte.10 PMC9632002 36824593

[41] Alonge M, Soyk S, Ramakrishnan S, Wang X, Goodwin S, Sedlazeck FJ, et al. RaGOO: fast and accurate reference-guided scaffolding of draft genomes. Genome Biol. 2019;20(1):1–17. doi: 10.1186/s13059-018-1612-0 31661016 PMC6816165

[42] Numa H, Itoh T. MEGANTE: a web-based system for integrated plant genome annotation. Plant Cell Physiol. 2013;55(1):e2–e2. doi: 10.1093/pcp/pct157 24253915 PMC3894707

[43] Solovyev V, Kosarev P, Seledsov I, Vorobyev D. Automatic annotation of eukaryotic genes, pseudogenes and promoters. Genome Biol. 2006;7(1):S10. doi: 10.1186/gb-2006-7-s1-s10 16925832 PMC1810547

[44] Braich S, Matchett-Oates L, Bailie B, Mohammaen E, Fulgueras K, Rochfort S, et al. Reference Genome and Whole Genome Resequencing in Medicinal Cannabis for Genomic Selection and Genome Editing, Enabling Precision Breeding. In San Diego; 2019.

[45] Li H. Aligning sequence reads, clone sequences and assembly contigs with BWA-MEM. arXiv Prepr arXiv13033997. 2013;

[46] Li H, Handsaker B, Wysoker A, Fennell T, Ruan J, Homer N, et al. The sequence alignment/map format and SAMtools. Bioinformatics. 2009;25(16):2078–9. doi: 10.1093/bioinformatics/btp352 19505943 PMC2723002

[47] Edgar RC. MUSCLE: multiple sequence alignment with high accuracy and high throughput. Nucleic Acids Res. 2004;32(5):1792–7. doi: 10.1093/nar/gkh340 15034147 PMC390337

[48] Labun K, Montague TG, Gagnon JA, Thyme SB, Valen E. CHOPCHOP v2: a web tool for the next generation of CRISPR genome engineering. Nucleic Acids Res. 2016;44(W1):W272–6. doi: 10.1093/nar/gkw398 27185894 PMC4987937

[49] Prykhozhij S V, Rajan V, Gaston D, Berman JN. CRISPR multitargeter: a web tool to find common and unique CRISPR single guide RNA targets in a set of similar sequences. PLoS One. 2015;10(3):e0119372. doi: 10.1371/journal.pone.0119372 25742428 PMC4351176

[50] Haeussler M, Schönig K, Eckert H, Eschstruth A, Mianné J, Renaud J-B, et al. Evaluation of off-target and on-target scoring algorithms and integration into the guide RNA selection tool CRISPOR. Genome Biol. 2016;17(1):148. doi: 10.1186/s13059-016-1012-2 27380939 PMC4934014

[51] Hwang WY, Fu Y, Reyon D, Maeder ML, Tsai SQ, Sander JD, et al. Efficient genome editing in zebrafish using a CRISPR-Cas system. Nat Biotechnol. 2013;31(3):227. doi: 10.1038/nbt.2501 23360964 PMC3686313

[52] Corporation GC. Sequencher verson 5.4.6 DNA sequence analysis software. Ann Arbor: Gene Codes Corporation; 2011.

[53] Booth JK, Page JE, Bohlmann J. Terpene synthases from Cannabis sativa. PLoS One. 2017;12(3):e0173911. doi: 10.1371/journal.pone.0173911 28355238 PMC5371325

[54] Walter MH, Hans J, Strack D. Two distantly related genes encoding 1‐deoxy‐d‐xylulose 5‐phosphate synthases: differential regulation in shoots and apocarotenoid‐accumulating mycorrhizal roots. Plant J. 2002;31(3):243–54. doi: 10.1046/j.1365-313x.2002.01352.x 12164805

[55] Seetang-Nun Y, Sharkey TD, Suvachittanont W. Molecular cloning and characterization of two cDNAs encoding 1-deoxy-d-xylulose 5-phosphate reductoisomerase from Hevea brasiliensis. J Plant Physiol. 2008;165(9):991–1002. doi: 10.1016/j.jplph.2007.06.014 17936410

[56] Kim S-M, Kuzuyama T, Kobayashi A, Sando T, Chang Y-J, Kim S-U. 1-Hydroxy-2-methyl-2-(E)-butenyl 4-diphosphate reductase (IDS) is encoded by multicopy genes in gymnosperms Ginkgo biloba and Pinus taeda. Planta. 2008;227(2):287–98. doi: 10.1007/s00425-007-0616-x 17763867

[57] Phillips MA, D’Auria JC, Gershenzon J, Pichersky E. The Arabidopsis thaliana type I isopentenyl diphosphate isomerases are targeted to multiple subcellular compartments and have overlapping functions in isoprenoid biosynthesis. Plant Cell. 2008;20(3):677–96. doi: 10.1105/tpc.107.053926 18319397 PMC2329938

[58] Bielecka M, Kaminski F, Adams I, Poulson H, Sloan R, Li Y, et al. Targeted mutation of Δ12 and Δ15 desaturase genes in hemp produce major alterations in seed fatty acid composition including a high oleic hemp oil. Plant Biotechnol J. 2014;12(5):613–23. doi: 10.1111/pbi.12167 24506492

[59] Grassa CJ, Weiblen GD, Wenger JP, Dabney C, Poplawski SG, Timothy Motley S, et al. A new Cannabis genome assembly associates elevated cannabidiol (CBD) with hemp introgressed into marijuana. New Phytol. 2021; doi: 10.1111/nph.17243 33521943 PMC8248131

[60] Braich S, Baillie RC, Jewell LS, Spangenberg GC, Cogan NOI. Generation of a comprehensive transcriptome Atlas and transcriptome Dynamics in Medicinal cannabis. Sci Rep. 2019;9(1):1–12. doi: 10.1038/s41598-018-37186-2 31719627 PMC6851104

[61] Springer NM, Ying K, Fu Y, Ji T, Yeh C-T, Jia Y, et al. Maize inbreds exhibit high levels of copy number variation (CNV) and presence/absence variation (PAV) in genome content. PLoS Genet. 2009;5(11):e1000734. doi: 10.1371/journal.pgen.1000734 19956538 PMC2780416

[62] Laverty KU, Stout JM, Sullivan MJ, Shah H, Gill N, Holbrook L, et al. A physical and genetic map of Cannabis sativa identifies extensive rearrangements at the THC/CBD acid synthase loci. Genome Res. 2019;29(1):146–56. 30409771 10.1101/gr.242594.118 PMC6314170

[63] Vergara D, Huscher EL, Keepers KG, Givens RM, Cizek CG, Torres A, et al. Gene copy number is associated with phytochemistry in Cannabis sativa. AoB Plants. 2019;11(6):plz074. doi: 10.1093/aobpla/plz074 32010439 PMC6986684

[64] McKernan KJ, Helbert Y, Kane LT, Ebling H, Zhang L, Liu B, et al. Sequence and annotation of 42 cannabis genomes reveals extensive copy number variation in cannabinoid synthesis and pathogen resistance genes. bioRxiv. 2020;

[65] Lavania UC. Genomic and ploidy manipulation for enhanced production of phyto-pharmaceuticals. Plant Genet Resour. 2005;3(2):170–7.

[66] Araki N, Kusumi K, Masamoto K, Niwa Y, Iba K. Temperature‐sensitive Arabidopsis mutant defective in 1‐deoxy‐d‐xylulose 5‐phosphate synthase within the plastid non‐mevalonate pathway of isoprenoid biosynthesis. Physiol Plant. 2000;108(1):19–24.

[67] Estévez JM, Cantero A, Reindl A, Reichler S, León P. 1-Deoxy-D-xylulose 5-phosphate, a limiting enzyme for plastidiciIsoprenoid biosynthesis in plants. J Biol Chem. 2001; doi: 10.1074/jbc.M100854200 11264287

[68] Stout JM, Boubakir Z, Ambrose SJ, Purves RW, Page JE. The hexanoyl‐CoA precursor for cannabinoid biosynthesis is formed by an acyl‐activating enzyme in Cannabis sativa trichomes. Plant J. 2012;71(3):353–65. doi: 10.1111/j.1365-313X.2012.04949.x 22353623

[69] Wang G, Dixon RA. Heterodimeric geranyl (geranyl) diphosphate synthase from hop (Humulus lupulus) and the evolution of monoterpene biosynthesis. Proc Natl Acad Sci. 2009;106(24):9914–9. doi: 10.1073/pnas.0904069106 19482937 PMC2701037

[70] Okada K, Kasahara H, Yamaguchi S, Kawaide H, Kamiya Y, Nojiri H, et al. Genetic evidence for the role of isopentenyl diphosphate isomerases in the mevalonate pathway and plant development in Arabidopsis. Plant Cell Physiol. 2008;49(4):604–16. doi: 10.1093/pcp/pcn032 18303110

[71] Xu C, Li H, Yang X, Gu C, Mu H, Yue Y, et al. Cloning and Expression Analysis of MEP Pathway Enzyme-encoding Genes in Osmanthus fragrans. Genes (Basel). 2016;7(10):78. doi: 10.3390/genes7100078 27690108 PMC5083917

[72] Hsieh M-H, Goodman HM. Functional evidence for the involvement of Arabidopsis IspF homolog in the nonmevalonate pathway of plastid isoprenoid biosynthesis. Planta. 2006;223(4):779–84. doi: 10.1007/s00425-005-0140-9 16231155

[73] Soyars CL, Peterson BA, Burr CA, Nimchuk ZL. Cutting Edge Genetics: CRISPR/Cas9 Editing of Plant Genomes. Plant Cell Physiol. 2018; doi: 10.1093/pcp/pcy079 29912402

